# Purification of Synthetic Gypsum: Techniques and Mechanisms

**DOI:** 10.3390/molecules31030484

**Published:** 2026-01-30

**Authors:** Can Wu, Wenting Xu, Zhizhao Song, Qingyun Ma, Qingjun Guan, Xuhui Qi, Xiaoya Li, Chengpeng Yang, Honghu Tang

**Affiliations:** 1 School of Resource Environment and Safety Engineering, Hunan University of Science and Technology, Xiangtan 411201, China; wucan522631@163.com (C.W.); xu_went@163.com (W.X.); s15350010090@outlook.com (Z.S.); 19044324748@163.com (Q.M.); 18073263718@163.com (X.Q.); lixy8878@126.com (X.L.); m16682559550@163.com (C.Y.); 2 Hunan Province Key Laboratory of Coal Resources Clean-Utilization and Mine Environment Protection, Xiangtan 411201, China; 3 School of Minerals Processing and Bioengineering, Central South University, Changsha 410083, China; honghu.tang@csu.edu.cn

**Keywords:** synthetic gypsum, purification, impurity, phase transformation, microbial method, high-value utilization

## Abstract

Synthetic gypsum (SG) is produced in massive quantities, yet hazardous impurities limit its reuse. This review summarized the impurity types in various SGs and the corresponding removal methods. Physical methods, such as washing, screening, magnetic separation, and others, exploit solubility and size/density differences to remove soluble salts and particulates. Chemical methods, including acid leaching, precipitation/solidification, and so on, can dissolve or immobilize phosphates, fluorides, and heavy metals. Flotation utilizes the differences in the physicochemical properties of solid surfaces to remove insoluble impurities. The thermal treatment is mainly used to decompose organics and improve whiteness. Microbial methods achieve environmentally friendly cleanup through metabolic leaching or microbially induced carbonate precipitation. The phase-transformation method is a recently developed method that can achieve synergistic effects of deep impurity removal and high-value utilization by reconstructing gypsum crystals to release co-crystallized impurities. Most impurity-removal methods target only a single type of impurity. At present, purifying SG requires a combination of multiple methods, which is not recommended from a cost perspective. Subsequent research on removing impurities from SG should focus on simultaneously removing multiple major impurities in a single process, as well as the synergistic effects between impurity removal and the high-value utilization of gypsum.

## 1. Introduction

Synthetic gypsum (SG), also known as chemical gypsum or industrial by-product gypsum, is a byproduct generated during industrial production. Its main component is calcium sulfate dihydrate (CaSO_4_·2H_2_O), along with various small amounts of impurities. The types of SG mainly include phosphogypsum (PG) [1] from the phosphate chemical industry, flue gas desulfurization (FGD) gypsum [2,3,4] from the flue gas desulfurization process, citrogypsum (CG) [5] from the citric acid industry, titanium gypsum (TG) [6] from the titanium dioxide industry, and salt gypsum (SAG) from the salt manufacturing process, among others [7,8,9]. Table 1 shows the annual production and utilization rate of different types of SG.

**Figure 1:**
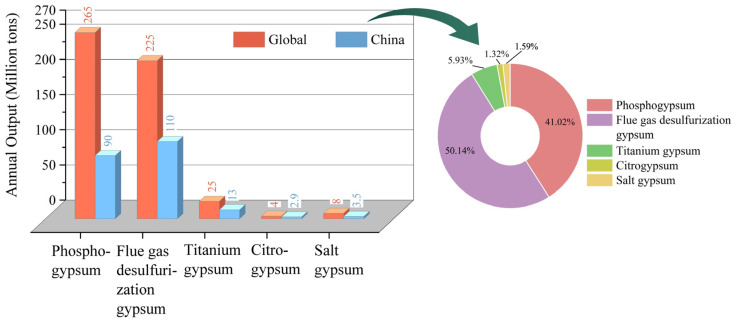
The annual production of different types of SG.

As shown in Figure 1, the global annual SG output ranges from 440 to 600 Mt, of which PG production exceeds 260 Mt annually [19]. In China, the annual production of SG is about 280 Mt. Due to the rapid expansion of coal-fired power plants, FGD gypsum has become the largest SG source, followed by PG [18]. The main component of SG is identical to that of natural gypsum, making it a promising alternative. However, only a small portion of SG is currently utilized effectively [20]. Among these, FGD gypsum, owing to its lower impurity content, achieves the highest utilization rate, up to 70%. The utilization rate of other SG stays below 40%, with some types dropping below 10% (as shown in Table 1). The remaining SG is usually stored or landfilled [21], of which PG stockpiles exceed 7000 Mt, with over 800 Mt in China [22]. During long-term storage, impurities in SG will be exposed to the environment through processes such as rainfall leaching and ecological cycling, potentially entering the human body and causing health risks. Moreover, Long-term stockpiling not only occupies land but also results in the loss of recoverable resources, such as rare earth elements in PG and iron or titanium in TG [23]. With growing public awareness of environmental protection and tightening government ecological policies, achieving stable, sustainable utilization of SG has become increasingly urgent. However, removing impurities is the primary step in solving this problem [24]. Relevant studies were identified through comprehensive searches of major scientific databases (e.g., Web of Science and ScienceDirect) using keywords. This review provides a systematic overview of impurity types in SG and a critical assessment of existing purification methods, highlighting their respective advantages and limitations and outlining future research directions.

## 2. Impurities and Their Hazards

The main components and impurities of various SG are summarized in Table 2 [25,26]. Different types of SG tend to contain different impurities due to variations in their raw materials and manufacturing processes, such as more phosphorus and fluorine impurities in PG and higher iron levels in TG (5–15%) [27]. However, SiO_2_ and Al_2_O_3_ are common impurities in all types of SG [23]. The presence of impurities limits the utilization of SG in various fields [28,29], as shown in Table 3. It is worth noting that PG may contain trace amounts of radionuclides (which are generally negligible in China). According to the European Union’s Basic Safety Standards Directive (2013/59/Euratom) and related guidelines, building materials are evaluated using the “gamma activity concentration index.” PG is explicitly classified as a construction material derived from radioactive residues and therefore requires radiological testing and assessment. When used in building applications, its activity index must ensure that the resulting exposure dose does not exceed the recommended reference level. The EU encourages the rational utilization of such materials, provided that the annual effective dose to the public remains within the prescribed limit, typically 1 mSv·year^−1^. Therefore, it is necessary to remove impurities for the SG recycling [30,31].

Impurities in SG can exist in several forms, including soluble impurities, surface-adsorbed species, insoluble impurities, and lattice-incorporated impurities. Soluble and surface-adsorbed impurities can generally be removed by simple washing. Insoluble impurities are typically introduced during mining as associated minerals and can be effectively separated by flotation. In contrast, lattice-incorporated impurities are formed through more complex mechanisms, mainly involving chemical reactions between gypsum and impurities or ionic substitution within the crystal lattice during industrial processing. Such impurities are difficult to remove by conventional methods and usually require phase transformation-based purification.

## 3. Impurity Removal Methods

### 3.1. Physical Methods

Physical methods utilize the differences in solubility, particle size, density, and magnetic properties between gypsum and impurities to separate them. Commonly used techniques include washing, sieving, classification, and magnetic separation.

#### 3.1.1. Washing

The washing removes soluble impurities from SG by dissolving them in water, making it a cost-effective and straightforward operation. This technique is frequently employed as a pretreatment step in SG purification. The process involves controlling the liquid-to-solid ratio and the number of washing cycles to transfer soluble impurities into the water, thereby reducing impurity contents in SG. The washing can efficiently eliminate water-soluble impurities such as NaCl, NaF, KF, P_2_O_5_, and some soluble radionuclides but is ineffective for poorly soluble compounds, including CaF_2_, fluorite, and fluorapatite [53]. It is worth noting that, owing to the slight solubility of gypsum and its hydration behavior in water, excessive washing may result in partial paste formation, thereby complicating handling and separation.

Water washing is primarily used to treat SAG to remove NaCl. Wang et al. [54] found that multi-stage countercurrent ultrasound washing enhanced removal efficiency. Under optimal three-stage ultrasonic countercurrent washing with a water-to-gypsum ratio of 5, the NaCl content in SAG decreased from 36,300 mg/kg to 75.7 mg/kg, corresponding to a 99.5% removal rate. In the case of PG, water washing is mainly used to eliminate residual acids and soluble impurities, such as phosphorus and fluorine. The number of washing cycles has a greater impact on fluorine removal from PG than the liquid-to-solid ratio. Typically, 4~5 cycles are necessary, with the ratio maintained between 3~4 [55]. Zhou et al. [56] applied a solid–liquid ratio of 2 to wash PG, increasing its pH from 1.75 to 5.00 with minimal water and removing over 80% of PO_4_^3−^, F^−^, and SO_4_^2−^. A lower initial pH requires additional washing cycles. While water washing is practical and straightforward, it achieves limited impurity removal and generates substantial volumes of wastewater.

#### 3.1.2. Sieving and Classification

Sieving and classification methods leverage differences in size and density to physically separate and remove impurities from SG. Sieving uses specific mesh sizes to classify gypsum particles by size, in either dry or wet conditions. Classification employs hydraulic settling or hydrocyclones to separate slurry based on settling velocity or density.

Wet sieving usually removes approximately 10~15% of coarse particle impurities from PG, leading to a notable decrease in insoluble impurity levels. For example, using a 300 μm vibrating screen for wet sieving effectively eliminates coarse particles rich in phosphorus and silicon, greatly improving gypsum purity [57]. Coarse particle impurities mainly consist of unreacted phosphate minerals, such as apatite, and co-crystallized fluoride and silicate compounds, including sodium fluorosilicate and fluorite, which make up over 98% of the total fluorine and silicon impurities in PG. Li et al. [58] used a 0.0308 mm sieve to remove fine-particle impurities, lowering the SiO_2_ content in PG from 14.11% to 4.49%, which represented a 31.8% removal rate. Particles smaller than 20 μm are highly enriched in ^226^Ra, ^210^Pb, Ba, U, and Th. Sieving effectively lowers the concentration of these radioactive elements in PG [59]. Hydraulic cyclone classification is suitable for slurry materials that cannot be screened directly. Other types of SG, such as FGD gypsum, CG, and SAG, can also be purified through sieving and classification when their impurities differ significantly in size or density from gypsum, allowing for effective separation [60].

The effectiveness of the methods mainly depends on how impurities are distributed across particle sizes. Significant purification is only achievable when impurities are concentrated in either coarse or fine particles; if impurities are evenly spread across all sizes, simple sieving or classification becomes ineffective. Economically, sieving and classification are simple, low-energy methods suitable for large-scale gypsum processing. They excel at removing particulate or insoluble impurities. However, they have limited effectiveness for eliminating impurities that are evenly distributed or chemically bonded, such as trace elements within the gypsum structure, which often require additional chemical purification techniques.

#### 3.1.3. Magnetic Separation

The magnetic separation method removes impurities from TG by leveraging differences in magnetic susceptibility between iron-containing impurities and nonmagnetic gypsum, enabling effective iron removal. Ding et al. [61] processed TG into a slurry and added a reducing agent, such as hydrogen, to convert cemented iron species into Fe_3_O_4_. This Fe_3_O_4_ was then separated from gypsum using magnetic equipment. The process is simple and continuous, achieving approximately 90% iron removal efficiency. The recovered Fe_3_O_4_ can be reused as a raw material for magnetic applications. Ma et al. [62] developed a combined process involving acid leaching, solvent extraction, and magnetic separation. In this process, iron impurities were leached from TG with HCl, followed by extraction using an organic extractant and acetone. Two-stage magnetic separation removed residual iron, and purified gypsum was obtained by precipitation and filtration, achieving about 90% iron removal. In summary, magnetic separation remains a feasible and efficient approach for removing high-iron impurities from SG.

### 3.2. Chemical Methods

Chemical methods remove impurities from SG by applying chemical reagents that cause dissolution, transformation, or separation. Common approaches include acid leaching, precipitation, and solidification, among others.

#### 3.2.1. Acid Leaching

Acid leaching is the most common chemical purification method. It serves two main purposes: first, it can convert poorly soluble impurities into soluble forms; second, it can break down the surface structure of impurities, helping them detach from the gypsum crystals and decompose. The main idea is to use solubility differences between impurities and gypsum: dissolve the impurities into the liquid, then separate the solid from the liquid. Reported acids for impurity removal include inorganic acids such as sulfuric, hydrochloric, and phosphoric acids, as well as organic acids such as citric, oxalic, boric, and malic acids. Acid leaching effectively removes phosphates, fluorides, rare earths, And radionuclides ^226^Ra and U (especially U(VI)) from PG [63,64]; iron from TG; and heavy metals from FGD gypsum [65,66,67].

Wang et al. [68] used acidic titanium dioxide wastewater (containing H_2_SO_4_ and Fe^2+^) for multi-stage leaching of TG, effectively removing iron and recycling the acid. Two acid leaching cycles at 70 °C for 2 h each achieved over 95% iron removal. Canovas et al. [53] leached heavy and light rare earth elements from PG using 0.5 M sulfuric acid, achieving overall leaching rates of about 58% and 46%, respectively, with especially high extraction efficiencies for Sc (99%) and Th (78%). This demonstrates the potential of PG as a secondary strategic resource for recovering rare earths, uranium, and other valuable elements. FGD gypsum, derived from coal-fired power plants, often contains trace levels of toxic elements such as arsenic and mercury, as well as unreacted alkaline residues. Li et al. [69] applied sulfuric acid leaching coupled with ultrasonic enhancement to address arsenic contamination, significantly improving arsenic removal efficiency. Using 8% H_2_SO_4_ for 60 min, arsenic leaching was 79.7% without ultrasound and increased to 97.09% with ultrasound. We [70] also examined arsenic removal from FGD gypsum produced by the nonferrous metal smelting industry and found that arsenic mainly exists in exchangeable forms. After leaching with H_2_SO_4_ at pH = 2, the residual forms become dominant, indicating that sulfuric acid leaching significantly decreases the exchangeable arsenic fraction. Inorganic acid treatments are highly effective at removing impurities; however, they produce acidic wastewater that requires additional treatment, increasing operational costs. Additionally, the strong acidity of these solutions can corrode equipment, leading to further technical and environmental challenges.

Recently, organic acid leaching has become an environmentally friendly method for purifying SG and removing impurities under mild conditions. For uses with less stringent quality standards, the products after treatment can often be used without further processing. Lin et al. [27] investigated the forms of iron impurities in TG and found that iron mainly existed as Fe(OH)_3_ on particle surfaces. Adding 10% citric acid at 80 °C, with a liquid-to-solid ratio of 8 and a reaction time of 80~90 min, achieved an iron removal efficiency of 84.37% and increased product whiteness from 8.1 to 36.5. Singh et al. [71] treated PG with 3~4% citric acid, showing that phosphate and fluoride impurities were converted into soluble phosphoric acid, hydrofluoric acid, and mixed salts, which were then removed by washing. This process greatly lowered the P_2_O_5_ and F contents in PG. Similarly, Cai et al. [67] reported that treating PG with 1% oxalic acid removed about 77.7% of P_2_O_5_, with the removal efficiency rising to 82.5% at 2% oxalic acid. Oxalic acid partly disrupted the gypsum crystal lattice, releasing trapped phosphate groups and effectively removing intercrystalline phosphorus. Despite these benefits, the relatively high cost of organic acids limits their practicality for large-scale industrial use.

#### 3.2.2. Precipitation and Solidification

Precipitation/solidification methods eliminate the adverse effects of impurities on gypsum by transforming soluble contaminants into insoluble precipitates or trapping them within stable matrices [72,73]. Specifically, appropriate reagents, such as alkaline residues or chemicals, are added to the gypsum slurry to react with impurities, such as phosphate, fluoride, and heavy metals, forming insoluble mineral phases (e.g., Ca_3_(PO_4_)_2_, CaC_2_O_4_, etc.). These precipitates can then be enclosed and stabilized by cement hydration products, such as calcium silicate hydrate (C–S–H) gels and ettringite, thus preventing the redissolution of hazardous species [74,75].

**Figure 2:**
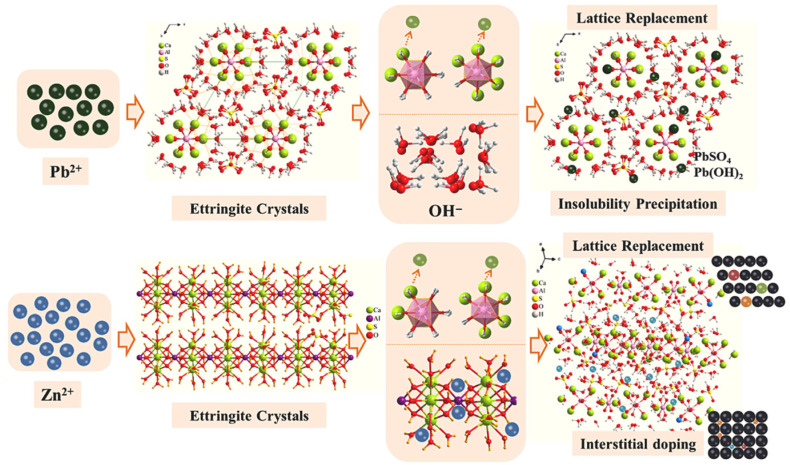
Mechanism of calcite solidification of heavy metals (Pb^2+^/Zn^2+^) [81]. Reproduced from *Cement and Concrete Research*, **2023**, *174*, 107350, with permission from Elsevier.

For PG, the primary challenge is the high level of soluble phosphorus and fluorine impurities. Adding alkaline reagents, such as lime, helps precipitate PO_4_^3−^ and F^−^ as stable crystalline compounds (calcium phosphate and calcium fluoride) [24]. Pretreating with Ca(OH)_2_, for instance, converts phosphate into CaHPO_4_·2H_2_O, reducing the soluble phosphorus content in PG by approximately 66–68% [76]. Similarly, adding alkaline industrial residues, such as carbide slag containing CaO, neutralizes PG acidity and significantly reduces fluoride levels. When about 3% of these residues are added, the concentrations of F^−^ and PO_4_^3−^ reach their lowest points [77]. Oxalic acid can convert insoluble phosphate in PG into soluble phosphorus, which are subsequently immobilized as stable calcium oxalate precipitates, highlighting its dual role in dissolution and solidification of impurities [78,79]. In the case of TG, the main impurities are Fe^3+^ and heavy metals. During hydration, Fe^3+^ forms Fe(OH)_3_ colloids that impede reactions. The addition of aluminum materials enables Fe^3+^ to react with aluminate ions, resulting in the formation of stable ferric ettringite phases such as Ca_6_[Al_11-x_Fe_x_(OH)_6_]_2_(SO_4_)_3_·26H_2_O [24]. Research indicates that co-sintering high-impurity TG with aluminosilicate wastes produced sulfoaluminate cement, which incorporated over 25% of TG impurities into clinker phases, primarily as ettringite. This cement exhibited high mechanical strength, with a 28 day compressive strength of 95.8 MPa [80]. Furthermore, heavy metals in TG were effectively stabilized within the calcium aluminate cementitious matrix, as illustrated in Figure 2 [81]. For example, the leaching retention rate of Cr reached 97.5%, and other heavy metals are almost undetectable in the leachate [80]. FGD gypsum generally exhibits high purity but may contain minor amounts of chloride ions. In cement systems, Cl^−^ can react with Ca^2+^ and Al^3+^ to form insoluble calcium chloroaluminate compounds, which facilitates effective immobilization. Due to its relatively low impurity contents, FGD gypsum can typically be used directly in building materials without significant loss of material performance [24].

Precipitation/solidification methods can be combined with slag and other industrial alkaline residues to balance SG valorization with cost and environmental benefits.

### 3.3. Flotation

Some impurities in SG exist as independent phases, as heterogeneous particles adsorbed on the gypsum surface, or embedded among its crystals. These impurities mainly come from unreacted minerals or external particulate matter, such as SiO_2_, Fe_2_O_3_, TiO_2_, Ca_3_(PO_4_)_2_, carbonaceous residues, and organic particles. Flotation is commonly used to remove this type of impurity, which separates impurities from gypsum by exploiting differences in the hydrophilicity/hydrophobicity of solid surfaces [82,83,84]. Existing studies primarily focus on removing quartz/siliceous phases, fine clays, and organics from PG.

**Figure 3:**
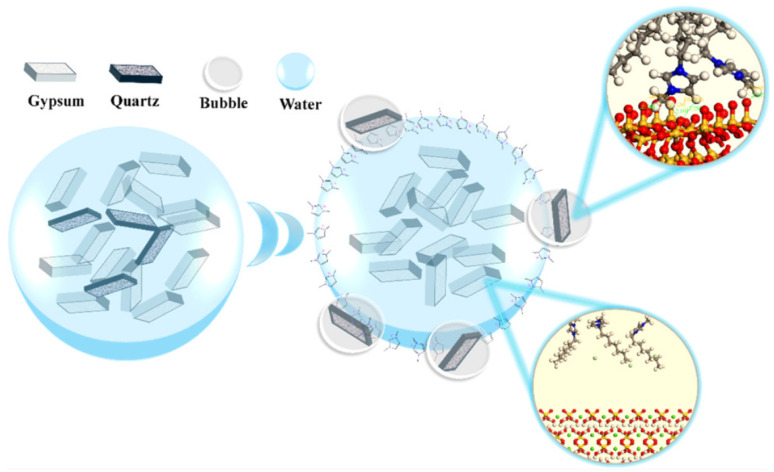
Flotation of quartz impurities from PG using collector 1-Methyl-3-Octylimidazolium chloride and schematic illustration [86]. Reproduced from *Applied Surface Science*, **2025**, *727*, 138208, with permission from Elsevier.

Since quartz and gypsum have similar surface properties, specialized collectors are necessary in selective flotation to differentiate their floatability [85]. Studies have shown that cationic collectors can substantially increase the hydrophobicity of quartz relative to gypsum. For instance, Yang et al. [86] employed 1-methyl-3-octylimidazolium chloride, achieving quartz recovery rates over 67% while keeping gypsum recovery below 6.5%, demonstrating high selectivity. The collector preferentially adsorbed onto the silicate surface of quartz through electrostatic attraction and hydrogen bonding, while on the gypsum surface, it interacted more weakly (Figure 3). Similarly, Shi et al. [87] reported that tetradecyl trimethyl ammonium chloride achieved quartz recovery up to 96% and gypsum below 25% under neutral conditions, significantly outperforming the traditional dodecylamine collector. Flotation also removes soluble impurities such as phosphorus and fluorine, which are transferred into the aqueous phase and are discharged with the froth; thus, the P_2_O_5_ and F contents in PG decrease notably after flotation [88]. However, flotation is less effective for removing lattice-bound phosphorus, such as “co-crystallized phosphorus.” In addition to improving purity, flotation also increases the whiteness of PG products. Fang et al. [85] reported that after two-stage flotation, the gypsum purity increased from 83.9% to 96.7%, SiO_2_ contents decreased to 0.07%, and product whiteness improved from 33.23 to 63.42. The removal of surface fine clays and organic matter during flotation changed PG from gray-black to a much lighter color.

Different collectors exhibit varying selectivity and operating conditions in flotation purification. For example, cetylpyridinium bromide at 10 mg/L and pH 5.5 achieved approximately 99% quartz recovery, while gypsum recovery was only 16% [89]. Tetradecyl trimethyl ammonium chloride showed optimal selectivity at near neutral pH [87]. Dodecyl dimethyl ethylbenzyl ammonium chloride, a quaternary ammonium salt containing an aromatic group, effectively removed SiO_2_, organic matter, and F^−^ simultaneously from acidic PG slurry, increasing the gypsum grade from 88% to over 94% [90]. 1-Methyl-3-octylimidazolium chloride enabled efficient quartz–gypsum separation without auxiliary agents, increasing the CaSO_4_·2H_2_O content in the PG concentrate to above 95% [86]. Each of these novel reagents has its own advantages and limitations: cetylpyridinium bromide and tetradecyl trimethyl ammonium chloride exhibit high selectivity but require careful pH control; dodecyl dimethyl ethylbenzyl ammonium chloride provides broader impurity removal but requires relatively higher dosages; imidazolium salts offer excellent selectivity at low doses but are more expensive. In contrast, traditional amine-based collectors show significantly poorer selectivity under similar conditions [87].

Flotation has shown relatively high efficiency, cost-effectiveness, and environmental compatibility in purifying PG. Through optimized flotation, impurities such as Si and F can be reduced to very low levels, raising the gypsum grade to over 95% and meeting the national standards for construction gypsum. However, flotation still has limitations in removing lattice-bound impurities. To achieve deeper purification and higher-value utilization, it must be combined with complementary treatment processes.

### 3.4. Phase Transformation

A key obstacle to improving the impurity-removal efficiency of SG is the incorporation of impurity ions into the CaSO_4_ crystal lattice, where they replace Ca^2+^ or SO_4_^2−^ sites. These tightly bound impurities, such as rare earth elements (La^3+^, Ce^3+^, Nd^3+^, etc.), Sr^2+^, Ba^2+^, F^−^, PO_4_^3−^, Al^3+^, Fe^3+^, As^5+^, Cr^6+^, and ^226^Ra, are difficult to dissolve or remove directly. To eliminate such strongly bound impurities, the phase transformation method is often employed [91]. This process removes impurities through a dissolution–recrystallization cycle that occurs during crystal phase changes (e.g., converting dihydrate gypsum to hemihydrate or anhydrite) [92,93,94]. During this process, the gypsum lattice disintegrates, releasing impurities as the original crystals dissolve [95]. Insoluble or co-crystallized impurities are released into the solution, allowing calcium and sulfate ions to recrystallize, while impurities stay in the mother liquor instead of reintegrating into the new crystal structure [96]. The main factors influencing impurity removal by the phase transformation method generally include three aspects: the acidic environment, the form in which impurity ions exist in the solution, and the crystal morphology and size of the recrystallized substance [97].

He et al. [98] achieved efficient removal of phosphorus impurities by converting PG to α-hemihydrate gypsum at 90 °C in 30% H_2_SO_4_. This process disrupted the gypsum lattice, releasing insoluble and intercrystalline phosphates and transforming them into soluble phosphoric species.

Another study employed a progressive purification approach [83]: first, SiO_2_ and Fe_2_O_3_ impurities in PG were removed using a silane coupling agent and tributyl phosphate, achieving removal rates of 98.5% for SiO_2_ and 95.5% for Fe_2_O_3_. Then, the dihydrate phase was transformed into anhydrite in a mixed H_2_SO_4_-NaCl solution, which effectively released and dissolved the co-crystallized phosphates, reducing total P to 0.04%, and enhanced the product whiteness to 92.5%.

**Figure 4:**
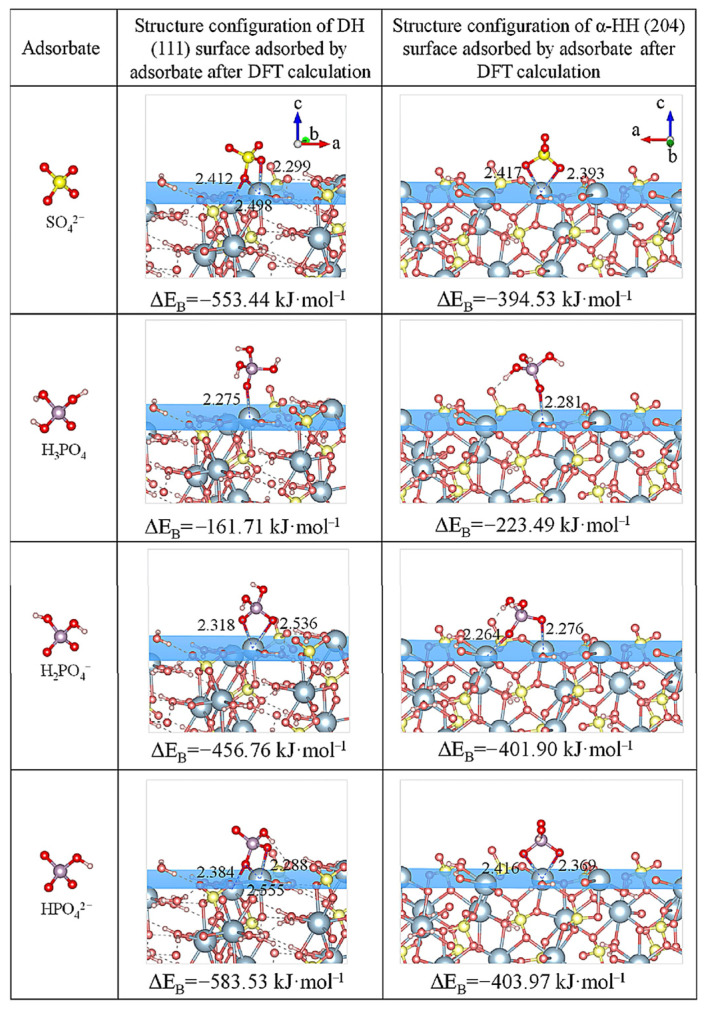
The structural configuration of the crystal surface after adsorption of the adsorbate, as obtained from density functional theory calculations [100]. Reproduced from *Minerals Engineering*, **2023**, *201*, 108203, with permission from Elsevier.

Our group investigated the mechanism of co-crystallized phosphorus removal from PG in salt-acid mixed solutions (including NaCl-HCl, Na_2_SO_4_-H_2_SO_4_, and CaCl_2_-HCl solution [97,99,100]. Figure 4 shows the structural configurations and binding energies of various adsorbates on the (204) surface of α-hemihydrate gypsum using DFT calculations. The calculated negative binding energy sequence was HPO_4_^2−^ > H_2_PO_4_^−^ >SO_4_^2−^ > H_3_PO_4_, indicating that under acidic conditions, phosphorus primarily exists as H_3_PO_4_, which is difficult to incorporate into the α-hemihydrate gypsum lattice through chemisorption or isomorphous substitution [100]. Under optimal mild conditions (90–95 °C), the phosphorus leaching rate exceeded 97%. Similarly, we observed the same pattern in the arsenic removal process of arsenic-containing FGD gypsum. During the gypsum phase transition, acids could effectively regulate arsenic speciation in solution, making it challenging to co-crystallize with the recrystallized substance [70]. In addition, some persulfate salts, such as K_2_S_2_O_8_, could also be used to regulate impurity species in solution, which were activated under hydrothermal conditions to produce sulfate radicals that oxidized impurity ions, thereby preventing the recombination of the released impurities with gypsum [91].

The formation of large-grained, nearly spherical crystals with low specific surface area and low surface activity during phase transformation can further assist in purifying gypsum [83,101]. We employed a seed-induced crystallization method to control the nucleation and growth of the recrystallized material. The introduction of preprepared α-type seeds with a specific shape induced the formation of large, uniform, short columnar α-hemihydrate gypsum, thereby reducing the product’s surface area and minimizing secondary adsorption of impurities [70]. Importantly, the large-grained, short-columnar α-hemihydrate gypsum exhibited substantial mechanical strength, making it a high-value-added gypsum product.

Phase transformation methods typically rely on acid–salt systems and elevated temperatures, resulting in relatively high chemical consumption and energy demand, which limits their applicability in low value-added systems. Meanwhile, these methods exhibit poor tolerance to high impurity contents. Despite these limitations, phase transformation methods can be effectively integrated with high value-added utilization routes, offering considerable potential for industrial application. We believe that integrating the phase-transition method with the hydrothermal process for producing α-hemihydrate gypsum could be a viable approach to achieving stable use of synthetic gypsum.

### 3.5. Heat Treatment

The heat treatment involves subjecting SG to high-temperature calcination, which induces dehydration and phase transformation of SG and facilitates the decomposition or stabilization of impurities. Studies have shown that under low-temperature calcination conditions (≈120 °C), P and F impurities in SG cannot be effectively removed and instead exhibit a relative enrichment due to the loss of crystal water [102]. As the calcination temperature increases to 400–500 °C, organic carbon in SG is almost completely decomposed, and silicate minerals are transformed into colorless SiO_2_ [8], thereby significantly improving the whiteness and purity of the gypsum; however, the removal efficiency for inorganic impurities such as P and F remains limited. At higher temperatures (600–800 °C), P and F are further converted into inert and insoluble compounds, including calcium pyrophosphate (Ca_2_P_2_O_7_), calcium metaphosphate (Ca(PO_3_)_2_), and calcium fluoride (CaF_2_). Although these impurities are not strictly removed, their chemical activity is substantially reduced, resulting in improved gypsum performance [103]. Notably, calcination above ~200 °C converts the gypsum to anhydrite rather than hydrated gypsum or hemihydrate, yielding a product with limited hydraulic reactivity. Overall, thermal treatment is effective in removing organic and carbonaceous contaminants and enhancing gypsum purity and whiteness. Still, it is less efficient for inorganic ions such as P and F. From both economic and environmental perspectives, the heat treatment is simple and does not generate secondary chemical pollution. However, it requires relatively high energy consumption.

### 3.6. Microbial Method

Microbial methods eliminate impurities from SG by producing microbial metabolic products, primarily via dissolution, adsorption, solidification, and reduction. It is primarily applied to the purification of PG, FGD gypsum, and TG, while the high-salinity environment of SAG is unfavorable for microbial viability.

#### 3.6.1. Microbial Dissolution

The microbial dissolution method eliminates impurities by utilizing microbial secretion of organic acids or enzymes, such as cellulase. These secretions decompose recalcitrant organic matter and activates insoluble impurities, resulting in their dissolution into the liquid. Representative microorganisms involved in this process include Aspergillus niger, Acidithiobacillus ferrooxidans, Acidiphilium cryptum, and Rhodotorula mucilaginosa [104,105,106]. A. niger, a typical phosphate-solubilizing fungus associated with, secretes substantial quantities of oxalic acid, which dissolves mineral phosphates. These bioacids increase solution acidity and facilitate impurity leaching. A. ferrooxidans, an autotrophic acidophilic bacterium, oxidizes Fe^2+^ to Fe^3+^ and forms jarosite, releasing protons and reducing the pH of the reaction system. This process generates a strongly acidic environment that favors the dissolution of poorly soluble elements, such as rare earth elements. Heterotrophic bacteria such as A. cryptum utilize organic carbon sources, such as glucose, to produce citric and other organic acids, thereby synergistically enhancing leaching efficiency [107]. The impurity removal effect of microbial dissolution method is greatly affected by bacterial species, reaction time and acidity control, and the treatment cycle is relatively long.

#### 3.6.2. Microbial Solidification

**Figure 5:**
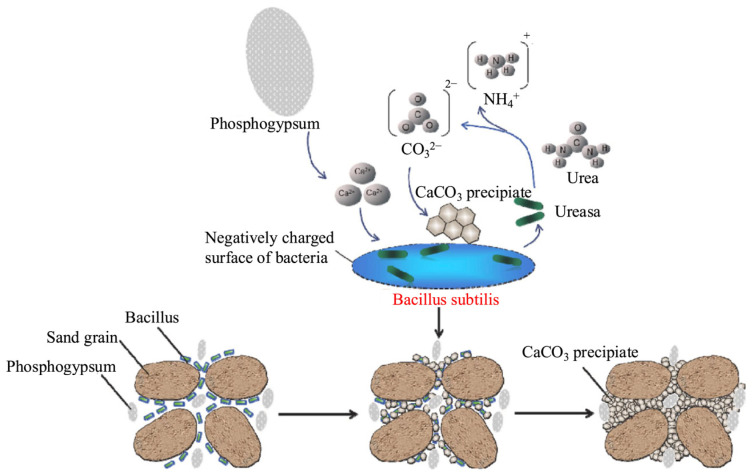
Schematic diagram of the principle of microbial-induced CaCO_3_ precipitation [110]. Reproduced from *Journal of Cleaner Production*, **2024**, *468*, 142999, with permission from Elsevier.

The microbial solidification method employs microorganisms or their metabolic products to induce mineral precipitation, immobilizing impurities such as P, F, and heavy metals in SG as stable mineral phases through coprecipitation or encapsulation [108]. Chen et al. [109] reported that R. mucilaginosa can generate PO_4_^3−^, which reacts with F^−^ and Ca^2+^ released from PG or TG to form stable fluorapatite, Ca_5_(PO_4_)_3_F, thereby significantly decreasing the bioavailability of phosphorus and fluorine. Another notable microbial solidification strategy is microbially induced carbonate precipitation and enzyme-induced carbonate precipitation (Figure 5) [110,111,112]. In these methods, urease-producing microorganisms or free urease catalyze urea hydrolysis to generate CO_3_^2−^, which subsequently reacts with Ca^2+^ to form CaCO_3_ precipitates, facilitating coprecipitation and mineralization of impurities. This method is usually accompanied by adsorption [113]. When metal ions in impurities (such as Cd^2+^, Zn^2+^, Sr^2+^, Pb^2+^) are divalent and have radii and hydration characteristics similar to those of Ca^2+^, they will replace part of the Ca^2+^ during CaCO_3_ growth, forming a solid solution or lattice doping [49,107]. Additional impurities, including P and F, can also be immobilized through coprecipitation, encapsulation, or adsorption as stable mineral phases [109,114]. These methods are geared towards stabilization and harmlessness rather than strict impurity removal and are suitable for reducing the environmental risks posed by harmful impurities and promoting their resource utilization.

#### 3.6.3. Microbial Reduction

**Figure 6:**
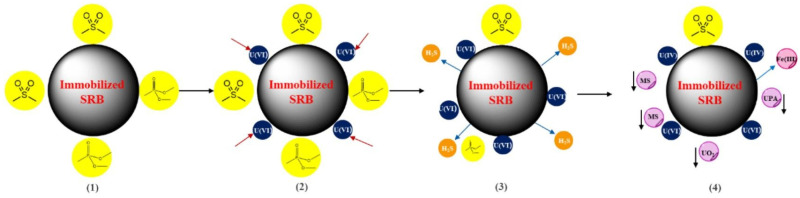
The mineralization mechanism of U(VI) by PG-SRB (PG-sulfate-reducing bacteria) [121]. Reproduced from *Chemical Engineering Journal*, **2024**, *493*, 152676, with permission from Elsevier.

The microbial reduction method uses microorganisms under anaerobic reducing conditions to convert impurities into low-valence, easily separable forms [114,115,116,117]. A typical mechanism involves sulfate-reducing bacteria reducing sulfate ions to sulfides, which then react with dissolved heavy metals to form stable metal sulfide precipitates [118,119]. Simultaneously, the metabolism of sulfate-reducing bacteria depletes the solution of acidity, significantly increasing the system pH and promoting pollutant stability. For iron-containing impurities, iron-reducing bacteria can reduce Fe^3+^ to low-valence, insoluble compounds, which are then removed by magnetic separation [120]. For uranium impurities, sulfate-reducing bacteria can reduce U(VI) to a sparingly soluble U(IV) and precipitate it [121] (Figure 6). Reduction methods are highly efficient at removing heavy metals, uranium, and some iron impurities. Still, they require strict anaerobic conditions and a specific carbon source, and the reaction rate is relatively slow.

## 4. Comparative Evaluation

The mechanisms, applicable impurity types, impurity removal efficiency, cost and resource consumption, environmental risks, process adaptability and secondary waste and treatment of all the above impurity-removal methods are summarized in Table 4.

From an overall engineering perspective, impurity-removal technologies for SG involve clear trade-offs among purification efficiency, cost, and environmental burden. Physical methods are the most mature and straightforward, with low capital and operating costs, and are well suited for removing soluble salts or impurities enriched in specific particle-size fractions. However, water washing is often accompanied by high water consumption and substantial wastewater generation. Chemical methods are generally effective in reducing phosphorus, fluorine, and certain heavy metals and exhibit broad applicability, but they require large amounts of reagents and tend to generate acidic or alkaline effluents and solid residues, leading to relatively high environmental risks and operating costs. Flotation performs reliably in removing quartz and organic impurities and can significantly improve gypsum purity and whiteness, showing good industrial applicability. Phase-transformation methods, based on dissolution–recrystallization processes, enable the efficient release and removal of co-crystallized impurities and offer clear advantages in terms of purification depth and product value enhancement; nevertheless, their overall cost and process management requirements are relatively high. Thermal treatment is effective for eliminating organic impurities and improving whiteness, but its application is largely constrained by high energy consumption and stringent flue-gas treatment requirements. Microbial methods feature the lowest resource and energy demands and minimal environmental impact; however, their long reaction times and limited process stability restrict large-scale engineering applications, making them more suitable as auxiliary or complementary options for risk reduction. In practice, a stepwise strategy is often preferred, in which low-cost physical separation or flotation is first applied, followed by chemical or phase-transformation treatments targeting refractory impurities, so as to achieve a balanced compromise between purification performance, cost, and environmental impact.

Most reported SG impurity-removal methods are still at the laboratory scale, and their industrial applicability is mainly constrained by cost, chemical and water consumption, equipment requirements, and secondary waste management. In practice, processes based on mature operations such as washing, classification, flotation, and acid leaching are generally considered more scalable. The large-scale utilization of FGD gypsum demonstrates that gypsum purification can be commercially viable when product quality is controlled through washing, dewatering, and standardized specifications. PG has also been utilized at an industrial scale by several enterprises; for example, phosphate chemical enterprises in China (e.g., Linhua Group) have removed impurities from PG via flotation and acid leaching and subsequently applied it in gypsum boards and other building materials. Similarly, TG has been preliminarily purified in some TiO_2_ plants through relatively simple processes such as water washing, neutralization, and dewatering, and has been used as a cement retarder and as a supplementary material in construction. These industrial practices indicate that TG is technically feasible for engineering applications, provided that acidity and soluble impurities are effectively controlled. In contrast, for SAG, the main barriers to scale-up lie in the removal and recovery of soluble salts, and industrially oriented processes typically emphasize brine recycling and wastewater minimization.

## 5. Conclusions

Presently, the biggest challenge in removing impurities from SG is that most impurity removal methods target only a single type of impurity. Removing all impurities requires a combination of multiple methods, which inevitably increases the cost of impurity removal. Additionally, purified gypsum remains a low-value product with limited applications. For example, industrially mature methods such as flotation and precipitation are relatively simple and cost-effective; however, they exhibit limited impurity removal efficiency and typically yield low-value dihydrate gypsum products, resulting in limited application potential. Subsequent research on removing impurities from SG should focus on simultaneously removing multiple major impurities in a single process, as well as the synergy between impurity removal and the high-value utilization of gypsum, which could be regarded as a short-term objective for impurity removal from SG. The phase transformation represents a promising approach for this process. This technique enables the conversion of low-value dihydrate gypsum into high-value α-hemihydrate or anhydrous gypsum while effectively removing co-crystalline impurities. α-Hemihydrate gypsum produced from SG can be directly applied in high-strength gypsum products (such as molding gypsum, precision casting molds, and ceramic and artistic molds), as well as in building and decorative materials (including high-strength gypsum boards and repair mortars). The process offers synergy between impurity removal and gypsum value enhancement. However, existing research primarily focuses on eliminating individual impurities and does not address the simultaneous elimination of multiple contaminants. Achieving synergistic removal of multiple major impurities during phase transformation could provide a stable, sustainable solution for SG recycling, such as the concurrent removal of phosphorus, fluorine, and black insoluble substances in a single step during the conversion of PG to high-value α-hemihydrate gypsum. Moreover, elucidating the microscopic occurrence and binding behavior of impurities, together with achieving low-cost and efficient impurity removal, can be regarded as long-term objectives for impurity removal from SG.

## Figures and Tables

**Table 1:** The origin, annual output, and utilization rate of different types of SG.

SG Types	SG Outputs per Ton of Product (t)	Utilization Rate (%)	References
PG	4~5	30~50	[10,11,12]
FGD gypsum	1~2.7	60~70	[13]
TG	6~12	10~30	[14]
CG	1.3~2.4	30~40	[15,16]
SAG	0.016~0.05	<10	[17,18]

**Table 2:** The chemical composition of different SG (mass %).

SG Types	pH	Moisture Content/%	Component Content/%										Ref.
			CaO	SO_3_	SiO_2_	P_2_O_5_	Fࢤ	Al_2_O_3_	Fe_2_O_3_	MgO	TiO_2_	MnO	
PG	1~4.5	20~30	32.80	44.40	1.37	1.69	0.55	0.11	0.03	0.01	-	-	[32]
			26.77	36.96	13.94	1.58	0.52	0.68	0.12	0.69	-	-	[25]
			33.65	34.00	4.74	1.82	0.30	2.30	1.38	0.08	-	-	[33]
FGD gypsum	6~8	15~20	50.80	42.70	2.00	0.02	0.42	1.10	0.64	1.06	0.10	0.60	[34]
			32.40	42.60	2.70	-	-	0.80	0.60	1.00	-	-	[35]
TG	3~6.5	20~30	33.90	36.70	4.10	-	-	1.20	13.00	0.90	8.50	-	[36]
			38.48	39.13	4.05	-	-	1.16	11.18	2.08	2.52	0.33	[27]
CG	2~3	20~40	39.28	55.88	0.83	-	-	0.15	0.24	0.5	-	-	[37]
			31.70	45.69	-	-	-	0.12	0.04	0.04	-	-	[38]
			43.36	55.47	0.54	0.08	-	0.13	-	0.06	-	-	[39]
SAG	7~8.5	15~30	22.2~36.6	40.7~44.7	-	-	-	-	-	-	-	-	[40]
			33.90	48.60	0.20	-	-	0.02	0.03	0.40	-	-	[41]

**Table 3:** Impurities in different types of SG and their hazards.

SG Types	Impurity Types	Content/%	Primary Form	Hazards and Application Limitations				Ref.
				Building Materials	Chemical Materials	Water Treatment	Agriculture	
PG	Phosphoric acid	0.5~3.5	H_3_PO_4_, H_2_PO_4_^−^, HPO_4_^2−^, PO_4_^3−^	① When phosphorus and fluorine exceed 0.3%, hydration products form coarser crystals, prolonging setting time and reducing strength; ② Organic materials coat gypsum crystals, increasing PG’s water demand when used as a building material; ③ High Chloride ions (Cl^−^) contents make gypsum boards absorb moisture, leading to mold and slow drying; ④ Iron impurities give gypsum a reddish-yellow color and can cause excessive hydration in cement-based materials; ⑤ Salt efflorescence leads to moisture absorption, surface crystallization, and corrosion of gypsum products.	① Impurities clog gypsum crystals, harming advanced materials like nanofibers; ② Excessive toxic metals, radioactive elements and others make products unsafe; ③ Although gypsum may exhibit corrosivity due to its relatively low pH, Cl^−^ can further accelerate corrosion, particularly in steel-containing systems; ④ High iron and impurity levels reduce the corrosion resistance of chemical products; ⑤ Residual organics may cause secondary pollution or catalyst poisoning; ⑥ High salinity hampers chemical reactions and lowers efficiency.	① Soluble phosphorus causes eutrophication in water bodies; ② Fine silica particles complicate water filtration; ③ Heavy metals migrate and pollute water bodies; ④ Organic matter increases the chemical oxygen demand (COD) of water, causing microbial growth; ⑤ Introducing large amounts of ions like Na^+^, Cl^−^, Mg^2+^, etc., increases TDS and salinity in water.	① Harmful impurities such as fluorine are toxic to bones, growth, and development in plants and animals; ② Insufficiently decomposed organic residues may lead to soil microbial contamination; ③ Elevated soil salinity can cause or worsen soil salinization, hindering crop growth.	[42,43,44]
	Phosphate		CaHPO_4_·2H_2_O, phosphate complex, apatite					
	Fluoride	0.06~1.66	F^−^, CaF_2_, CaSiF_6_, Na_3_AlF_6_					
	Organic matter	0.3~0.8	rotting plant/organism					
	Aluminosilicate	2~14	SiO_2_, Al_2_O_3_					
	Heavy metals	0.05~0.2	As, Cd, Cr, Hg, Pb, Fe					
	Radionuclides	0.05~0.2	UO,^226^Ra,^232^Th,^40^K					
FGD gypsum	Aluminosilicate	5~10	SiO_2_, Al_2_O_3_					[45,46,47]
	Heavy metals	0.01~0.05	As, Cd, Cr, Hg, Pb, Fe					
	Chlorine	0.01~0.35	Cl-					
	Fluoride	0.01~0.05	F^−^, CaF_2_					
TG	Titanium	2.5~8.5	TiO_2_					[48,49]
	Aluminosilicate	5~10	SiO_2_, Al_2_O_3_					
	Fluoride	0.01~0.05	H_2_SiF_6_, CaF_2_					
	Heavy metals	11~23	As, Cd, Cr, Hg, Pb, Fe					
CG	Organic matter	0.1–0.3	Na_3_C_6_H_5_O_7_, C_6_H_8_O_7_, Organophosphate					[50,51]
	Inorganic compounds	3.0~8.0	CaC_2_O_4_, P_2_O_5_					
SAG	Salts	3.0~6.0	NaCl, CaCO_3_, MgCO_3_, K_2_SO_4_, MgCl_2_					[52]

**Table 4:** Comparative evaluation of different impurity removal methods for SG in terms of efficiency, cost, environmental risk, and process adaptability.

Methods		Mechanisms	Target Impurities	Impurity Removal Efficiency	Cost and Resource Consumption	Environmental Risks	Process Adaptability	Secondary Waste and Treatment
Physical methods	Water washing	Dissolving water-soluble salt impurities in water for solid–liquid separation.	Soluble phosphorus, fluoride, chlorides, sulfates, phosphates, etc.	F^−^, PO_4_^3−^, Cl^−^ ≈ 60~70%	High water consumption, low cost.	Medium	Widely applicable, can be industrialized.	Wastewater needs to be treated, Cl^−^ may exceed the standard.
	Screening/ classification	Separating impurities by size or density.	Unreacted minerals in coarse particles; fluorine- or silicon-bearing compounds; fine clays; radioactive particulates, etc.	SiO_2_, ^226^Ra, Enriched particles ≈ 20~40%	Low energy consumption, low cost.	Low	Widely applicable, impurity particle size enrichment is required, simple equipment, can be industrialized.	Dust needs to be controlled.
	Magnetic separation	Separates iron impurities based on the magnetic differences between substances.	Iron oxides and other Fe-bearing impurities (mainly in TG).	Fe_2_O_3_ ≥ 85%	Medium energy consumption, requires reducing agent, medium cost.	Low	Suitable for TG with high Fe impurities, simple equipment, can be industrialized.	Almost no waste liquid, waste gas, or secondary waste.
Chemical methods	Acid leaching	Using acid to dissolve impurities into the soluble phase or precipitable compounds.	Sparingly soluble impurities, such as alkaline oxides, phosphates, and some heavy metals.	P, F^−^, heavy metal ≈ 60~99%	Large acid consumption, medium to high cost.	Low	Widely applicable, industrialization requires corrosion prevention, simple equipment, can be industrialized.	It generates waste liquid containing phosphorus, phosphorus, heavy metals, etc., which is difficult to treat.
	Precipitation/ solidification	Using alkaline substances to precipitate impurities into cementitious or stable matrices.	Phosphorus and fluorine impurities, iron and aluminum compounds, heavy metals, etc.		Low resource consumption, low cost.	Medium	Widely applicable, can be industrialized.	The resulting sludge contains CaF_2_, phosphates, and other substances, which require landfill disposal.
Flotation		Using mineral surface hydrophobicity differences to separate impurities from gypsum via froth flotation.	Insoluble independent phase or heterogeneous particles, such as quartz, carbonaceous residues, and organic matter.	SiO_2_, C, organic matter > 90%; F^−^ ≈ 20~40%	Medium chemical consumption, low energy consumption, medium cost	Medium	Widely applicable, simple equipment, can be industrialized.	The flotation wastewater produces tailings containing reagents and circulating water, making it difficult to treat.
Phase transformation		Converting dihydrate gypsum to hemihydrate or anhydrite to release lattice-bound impurities	Lattice-incorporated impurities (co-crystallized phosphorus, heavy metals, rare earth elements, etc.).	Co-crystallized P, F, REEs > 95%	Salt-acid consumption is medium, heating is required, medium to high cost.	High	Widely applicable, industrialization prospects depend on cost control.	The waste liquid contains high concentrations of phosphates, sulfates, and large amounts of heavy metals, requiring special treatment.
Heat treatment		Controlling temperature and atmosphere to facilitate crystal reconstruction and impurity changes, leading to impurity decomposition, migration, or immobilization.	Organic impurities, organic carbon, volatile phosphorus–fluorine precursors, etc.	Organic impurities ≈ 100%, P, F ≈ 40~60%	Needs to be heated to high temperature, high energy consumption, high cost.	Medium	Widely applicable, but high costs limit large-scale promotion.	Toxic substances are reduced to gaseous pollutants and require treatment.
Microbiological methods	Microbial dissolution	Microorganisms release organic acids and enzymes that facilitate the decomposition of insoluble impurities.	Phosphates, rare earths, etc.	P, F, heavy metal 50~80%	No chemical reagents are consumed, but microorganisms need to be cultured, medium cost.	Low	Low applicability, limited applicability to extremely acidic gypsum, microbial control is difficult, industrialization is difficult.	Almost no waste liquid or waste gas, and the small amount of biological residue is easy to treat.
	Microbial solidification	Microorganisms produce urease and promote mineralization and precipitation	Phosphate, fluoride ions and some heavy metals.					
	Microbial reduction	Reducing bacteria convert impurities into low-valence state, easily separated forms.	Heavy metals, uranium, iron.					

## Data Availability

The original contributions presented in this study are included in the article. Further inquiries can be directed to the corresponding author.
